# Supplementary material for: The Adsorption of Small Molecules on the Copper Paddle-Wheel: Influence of the Multi-Reference Ground State
Source: Molecules. 2022 Jan 28;27(3):912. doi: 10.3390/molecules27030912 (PMC8840508; doi:10.3390/molecules27030912)
Supplement: Supplementary file 1 [file molecules-27-00912-s001.zip › Supplementary-molecules-1559927_Corrected.pdf]

Supplementary Materials: The adsorption of small molecules on the copper paddle-wheel: Influence of the multi-reference ground state

Marjan Krstić [1], Karin Fink [2] and Dmitry Sharapa [3,*]

1. Theoretical Methods

1.1. Density Functional Theory

All molecular structures were first optimized in Gaussian16[1] code with each DFT functional (PBE[2], B3LYP[3], M06L[4]) in both ground state and higher-spin state. For GGA and hybrid functionals additional Grimme's dispersion correction (D3)[5] with Becke-Johnson damping[6] was employed (D3BJ option). All calculations utilized Ahlrichs *et al.*[7] triple-ζ-valence-plus-polarization quality (def2-TZVP) basis set. The standard "Ultrafine" grid was used. To find correct electronic state we used option "Guess=Mix" to mix HOMO and LUMO to destroy α-β and spatial symmetries. After local minima were confirmed by performing vibrational analysis, additional single-point calculations with "Stable=opt" were made to re-optimize the wavefunction in the case of instability to produce correct energy of each system. The binding energies (E_b) were calculated as a difference of total energy of adsorbed molecules on the PW and sum of paddle-wheel energy and educt energy in the infinite separation for the ground electronic state. The factor of 27.2113862459885 was used to present binding energies in eV, rounded to the two decimal places.

1.2. Single- and multi-reference methods

Sample of input for DLPNO-MR-CCSD calculations:

```
! def2-TZVP def2-TZVP/C
! TightSCF
%pal nprocs 24 end
%base "AB"        # any name can be given here, but should be consistent with next job
%casscf
nel 2        # number of active electrons
norb 2        # number of active orbitals
mult 1        # multiplicity blocks
nevpt2 3        #DLPNO-NEVPT2=3, PC-NEVPT2=2, SC-NEVPT2=1
trafostep ri        # using RI approximation
DoDumpForMRCC 1
end
%MaxCore 20000
* xyz 0 1
  Cu  -0.003451000   0.002485000   1.172521000
  Cu  -0.003751000  -0.000328000  -1.401843000
  O   -1.966134000   0.007369000   1.207559000
  O   -0.008318000  -2.003890000  -1.212917000
  O   -0.008339000  -1.981731000   1.075536000
  O    2.029542000  -0.004580000  -1.075729000
  O   -2.036966000   0.004610000  -1.075284000
  O    0.000850000   2.003637000  -1.217077000
  O    1.959241000  -0.002303000   1.207130000
  O    0.001416000   1.986489000   1.071424000
  C   -0.009615000  -2.530366000  -0.064841000
  C    0.002417000   2.532624000  -0.070155000
  C    0.668325000  -0.004578000  -3.771765000
  C    2.534901000  -0.004710000   0.079113000
  C   -2.542057000   0.007284000   0.079673000
  C   -0.676296000  -0.001073000  -3.771632000
```

```
  H    1.237212000  -0.935910000  -3.781979000
  H    1.242063000   0.923749000  -3.783959000
  H   -1.250036000  -0.929425000  -3.781737000
  H   -1.245185000   0.930236000  -3.783714000
  H   -3.650764000   0.009983000   0.122986000
  H    0.005085000   3.641930000  -0.056756000
  H    3.643619000  -0.007316000   0.122176000
  H   -0.012283000  -3.639641000  -0.049010000
*

$new_job

! UHF DLPNO-CCSD def2-TZVP def2-TZVP/C TightSCF
! TightPNO MORead noiter
%moinp "AB.gbw"
%pal nprocs 16 end
%mdci
inname "AB"
mrcc on
mrcctype mkcc
n_docc 82
refs "20,02"
end
%Maxcore 75000
* xyz 0 1
  Cu  -0.003451000   0.002485000   1.172521000
  Cu  -0.003751000  -0.000328000  -1.401843000
  O   -1.966134000   0.007369000   1.207559000
  O   -0.008318000  -2.003890000  -1.212917000
  O   -0.008339000  -1.981731000   1.075536000
  O    2.029542000  -0.004580000  -1.075729000
  O   -2.036966000   0.004610000  -1.075284000
  O    0.000850000   2.003637000  -1.217077000
  O    1.959241000  -0.002303000   1.207130000
  O    0.001416000   1.986489000   1.071424000
  C   -0.009615000  -2.530366000  -0.064841000
  C    0.002417000   2.532624000  -0.070155000
  C    0.668325000  -0.004578000  -3.771765000
  C    2.534901000  -0.004710000   0.079113000
  C   -2.542057000   0.007284000   0.079673000
  C   -0.676296000  -0.001073000  -3.771632000
  H    1.237212000  -0.935910000  -3.781979000
  H    1.242063000   0.923749000  -3.783959000
  H   -1.250036000  -0.929425000  -3.781737000
  H   -1.245185000   0.930236000  -3.783714000
  H   -3.650764000   0.009983000   0.122986000
  H    0.005085000   3.641930000  -0.056756000
  H    3.643619000  -0.007316000   0.122176000
  H   -0.012283000  -3.639641000  -0.049010000
*
```

Calculations of the complexes are more expensive (RAM) than of the dissociated systems (adsorbate moved 50Å off). Typical cost of calculation listed above with usage of node with 1.5TB RAM node (and local scratch to speed up I/O) is 7-10 days (walltime, 16 cores used in second part).

```
           ***************************************************************
           *        Program running with 8 parallel MPI-processes        *
           *              working on a common directory                  *
           ***************************************************************
------------------------------------------------------------------------------
                           ORCA GTO INTEGRAL CALCULATION
------------------------------------------------------------------------------

                         BASIS SET STATISTICS AND STARTUP INFO

 # of primitive gaussian shells          ...  174
 # of primitive gaussian functions       ...  366
 # of contracted shells                  ...   82
 # of contracted basis functions         ...  194
```

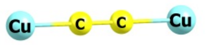


```
---------------------------------------------
CAS-SCF STATES FOR BLOCK  1 MULT= 1 NROOTS= 1
---------------------------------------------

ROOT   0:  E=   -3582.1954308656 Eh
      0.58356 [     0]: 20
      0.41644 [     2]: 02

Total time spent in the PNO generation part:       15.105 sec
 Total size for 2-ext PNO integrals ...     12.3 MB
--------------------------------
LOCAL RI TRANSFORMATION (VABPAO)
--------------------------------

Number of PAOs:     196
Basis functions:    194 (82 shells)
Aux. functions:     726 (218 shells)
```

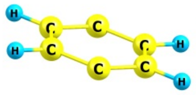


```
   IBatch   1 (of   2)               ... done (   31.689 sec)
   IBatch   2 (of   2)               ... done (   18.771 sec)
Total EXT                            ...          50.460 sec

---------------------
RI-PNO TRANSFORMATION
---------------------

Total Number of PNOs                    ... 28799
Total Number of IJ-PNOs                 ... 24893
Total Number of IP-PNOs                 ...  3768
Total Number of PQ-PNOs                 ...   138
Total Number of IJ-pairs                ...   664
Total Number of PI-pairs                ...    52
Total Number of PQ-pairs                ...     2
Average number of PNOs per pair         ...    40
Maximal number of PNOs per pair         ...    84
>> Statistics for ij-pairs..

Timings:
Total PNO integral transformation time     ...         36.370 sec
Size of the 3-external file                ...       444 MB
Size of the 4-external file                ...      4024 MB
Size of the IKJL file                      ...         4 MB
Size of the all 3-external file            ...      3586 MB
Size of the 1-external file                ...         6 MB

Size of the PAO/PNO coefficient matrices   ...      23.3 MB

Making pair/pair overlap matrices          ... done (    5.790 sec)
Total # unrestricted pairs ...       1398
  # of alpha-alpha pairs   ...        340
  # of alpha-beta pairs    ...        718
  # of beta-beta pairs     ...        340
Constructing the guess amplitudes          ... done (    0.201 sec)

-------------------------
FINAL STARTUP INFORMATION
-------------------------

E(0)                                       ...  -3582.008211401
Initial E(tot)                             ...  -3582.008211401
Number of pairs included                   ... 1398
----------------------------------------------------------
            DLPNO MR COUPLED CLUSTER ITERATIONS
----------------------------------------------------------

Number of PNO amplitudes to be optimized   ...      2365903
Number of non-PNO amplitudes               ...     26623512
Untruncated number of regular amplitudes   ...     14111604

Sigma for iref 0 Iter 0 : 222.851261 seconds
Sigma for iref 1 Iter 0 : 224.084391 seconds
```

```
           ***************************************************************
           *        Program running with 8 parallel MPI-processes        *
           *              working on a common directory                  *
           ***************************************************************
------------------------------------------------------------------------------
                           ORCA GTO INTEGRAL CALCULATION
------------------------------------------------------------------------------

                         BASIS SET STATISTICS AND STARTUP INFO

 # of primitive gaussian shells          ...  180
 # of primitive gaussian functions       ...  360
 # of contracted shells                  ...   88
 # of contracted basis functions         ...  196

---------------------------------------------
CAS-SCF STATES FOR BLOCK  1 MULT= 1 NROOTS= 1
---------------------------------------------

ROOT   0:  E=   -3691.4234392416 Eh
      0.52662 [     0]: 20
      0.47338 [     2]: 02
```

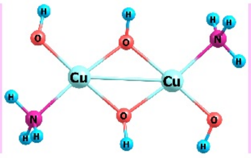


```
Total time spent in the PNO generation part:       32.136 sec
 Total size for 2-ext PNO integrals ...     45.5 MB
--------------------------------
LOCAL RI TRANSFORMATION (VABPAO)
--------------------------------

Number of PAOs:     198
Basis functions:    196 (88 shells)
Aux. functions:     714 (222 shells)

   IBatch  26 (of  27)               ... done (   59.442 sec)
   IBatch  27 (of  27)               ... done (   64.561 sec)
Total EXT                            ...        1390.207 sec

---------------------
RI-PNO TRANSFORMATION
---------------------

Total Number of PNOs                    ... 88217
Total Number of IJ-PNOs                 ... 78996
Total Number of IP-PNOs                 ...  9092
Total Number of PQ-PNOs                 ...   129
Total Number of IJ-pairs                ...  1540
Total Number of PI-pairs                ...   160
Total Number of PQ-pairs                ...     2
Average number of PNOs per pair         ...    51
Maximal number of PNOs per pair         ...   114
>> Statistics for ij-pairs..

Timings:
Total PNO integral transformation time     ...        470.922 sec
Size of the 3-external file                ...      1803 MB
Size of the 4-external file                ...     18355 MB
Size of the IKJL file                      ...        44 MB
Size of the all 3-external file            ...     37866 MB
Size of the 1-external file                ...        43 MB

Size of the PAO/PNO coefficient matrices   ...     133.2 MB

Making pair/pair overlap matrices          ... done (  301.455 sec)
Total # unrestricted pairs ...       3362
  # of alpha-alpha pairs   ...        830
  # of alpha-beta pairs    ...       1702
  # of beta-beta pairs     ...        830
Constructing the guess amplitudes          ... done (    1.128 sec)

-------------------------
FINAL STARTUP INFORMATION
-------------------------

E(0)                                       ...  -3691.009742750
Initial E(tot)                             ...  -3691.009742750
Number of pairs included                   ... 3362
----------------------------------------------------------
            DLPNO MR COUPLED CLUSTER ITERATIONS
----------------------------------------------------------

Number of PNO amplitudes to be optimized   ...      8841633
Number of non-PNO amplitudes               ...     64025928
Untruncated number of regular amplitudes   ...     17196732

Sigma for iref 0 Iter 0 : 2123.945653 seconds
Sigma for iref 1 Iter 0 : 2138.287763 seconds
```

Figure S1. Comparison of test DLPNO-MR-CCSD/def2-SVP calculations of Cu-C≡C-Cu + *m*-benzyne (C_6H_4) (left) and bis-μ-oxo $Cu_2O_2(OH)_2(NH_3)_2$ with active orbitals on Cu atoms (right)

Table S1. Overview of the complete set of DFT calculations from Gaussian16 code for ground-state and higher-spin state of each adsorbed educt. Gray rows label system models in the electronic ground state.

System	PBE+D3BJ S^2	PBE+D3BJ E[Hartree]	PBE+D3BJ E_b[eV]	B3LYP+D3BJ S^2	B3LYP+D3BJ E[Hartree]	B3LYP+D3BJ E_b[eV]	M06L S^2	M06L E[Hartree]	M06L E_b[eV]
PW	2.0000	-4194.06868643		2.0000	-4195.68495958		2.0000	-4195.326509	
	0.0161	-4194.07162811		0.0420	-4195.68622333		0.0366	-4195.32870644	
H_2O	0.0000	-76.3771034313		0.0000	-76.463569238		0.0000	-76.4432709964	
PW+H_2O	2.0000	-4270.46574519		2.0000	-4272.16994011		2.0000	-4271.79168569	
	0.0165	-4270.46857359	0.54	0.0405	-4272.1711197	0.58	0.0367	-4271.79376423	0.59
PW+2H_2O	2.0000	-4571.04662527		2.0000	-4573.05003082		2.0000	-4572.653928	
	0.0171	-4346.86455039	1.05	0.0389	-4348.65518221	1.14	0.0368	-4348.25778305	1.16
CO_2	0.0000	-188.48017332		0.0000	-188.671580523		0.0000	-188.653199027	
PW+CO_2	2.0000	-4382.55767536		2.0000	-4384.36752043		2.0000	-4383.99026477	
	0.0160	-4382.56060141	0.24	0.0413	-4384.36876187	0.30	0.0364	-4383.9924265	0.29
PW+2CO_2	2.0000	-4571.04662527		2.0000	-4573.05003082		2.0000	-4572.653928	
	0.0159	-4571.04953854	0.48	0.0405	-4573.05125282	0.60	0.0360	-4572.6560739	0.57
CO	0.0000	-113.234702047		0.0000	-113.363253396		0.0000	-113.34385415	
PW+CO	2.0000	-4307.31747929		2.0000	-4309.06034603		2.0000	-4308.68459401	
	0.0131	-4307.3212869	0.41	0.0420	-4309.06159096	0.33	0.0378	-4308.68697092	0.39
PW+2CO	2.0000	-4420.56392127		2.0000	-4422.43485113		2.0000	-4422.04084287	
	0.0184	-4420.56725439	0.71	0.0416	-4422.43606619	0.64	0.0400	-4422.0431371	0.73
O_2	2.0000	-150.249128478		2.0000	-150.390218301		2.0001	-150.369530084	
PW+O_2	2.0114	-4344.32697329	0.17	2.0284	-4346.08109718	0.13	2.0279	-4345.70482373	0.18
	6.0000	-4344.32415127		6.0001	-4346.07986258		6.0001	-4345.70288885	
PW+2O_2	6.0100	-4494.58130057	0.31	6.0247	-4496.47570578	0.25	6.0253	-4496.08003285	0.33
	12.0000	-4494.57931345		12.0001	-4496.47472194		12.0002	-4496.07914858	
C_2H_4	0.0000	-78.5027079242		0.0000	-78.6277014958		0.0000	-78.6021244651	
PW+C_2H_4	2.0000	-4272.58697385		2.0000	-4274.3288801		2.0000	-4273.94465349	
	0.0155	-4272.59008613	0.43	0.0411	-4274.33011157	0.44	0.0363	-4273.94683322	0.44
PW+2C_2H_4	2.0000	-4351.10253418		2.0000	-4352.97104125		2.0000	-4352.56039381	
	0.0164	-4351.10554779	0.78	0.0402	-4352.97225797	0.83	0.0365	-4352.56256202	0.81
MeOH	0.0000	-115.631483301		0.0000	-115.778780597		0.0000	-115.748928277	
PW+MeOH	2.0000	-4309.7127709		2.0000	-4311.47685395		2.0000	-4311.08794664	
	0.0160	-4309.72333799	0.55	0.0415	-4311.48534062	0.55	0.0363	-4311.099625	0.60
PW+2MeOH	2.0000	-4425.35656699		2.0000	-4427.26853921		2.0000	-4426.8489273	
	0.0159	-4425.37346723	1.06	0.0409	-4427.28797941	1.20	0.0375	-4426.8686354	1.14
EtOH	0.0000	-154.909528533		0.0000	-155.11658654		0.0000	-155.075242357	
PW+EtOH	2.0000	-4349.00033493		2.0000	-4350.82702858		2.0000	-4350.42603944	
	0.0165	-4349.00319532	0.60	0.0403	-4350.82820056	0.69	0.0367	-4350.42805112	0.66
PW+2EtOH	2.0000	-4503.92439702		2.0000	-4505.95881148		2.0000	-4505.52378	
	0.0160	-4503.93308023	1.15	0.0385	-4505.96863485	1.34	0.0369	-4505.52567813	1.26

Table S2. Total and relative energies related to Figure 3.(Dicopper tetraformate)

Method	C_2H_4			CO				
	$PW\text{-}C_2H_4$	$PW\cdot\cdot C_2H_4$	E_b[eV]	OC-PW-CO	OC··PW-CO	OC··PW··CO	$E1_b$[eV]	$E2_b$[eV]
HF	-4109.23255	-4109.22331	-0.25	-4256.73447	-4256.72264	-4256.72765	0.14	-0.32
PBE-D3BJ	-4115.45895	-4115.44159	-0.47	-4263.43708	-4263.42511	-4263.40828	-0.46	-0.33
B3LYP-D3BJ	-4116.95146	-4116.93481	-0.45	-4265.05486	-4265.04056	-4265.03329	-0.20	-0.39
M06-L	-4116.61481	-4116.59730	-0.48	-4264.71126	-4264.69706	-4264.68292	-0.38	-0.39
CASSCF	-4109.22592	-4109.21649	-0.26	-4256.72796	-4256.71600	-4256.72083	0.13	-0.33
DCD-CAS(2)	-4113.21146	-4113.19034	-0.57	-4261.10568	-4261.08805	-4261.07966	-0.23	-0.48
NEVPT2	-4113.21130	-4113.19030	-0.57	-4261.10540	-4261.08769	-4261.07930	-0.23	-0.48
MP2	-4113.21528	-4113.19438	-0.57	-4261.10911	-4261.09151	-4261.08340	-0.22	-0.48
SOS-MP2	-4112.92839	-4112.91143	-0.46	-4260.79281	-4260.77703	-4260.77276	-0.12	-0.43
MP3	-4112.64656	-4112.62799	-0.51	-4260.50663	-4260.48991	-4260.48597	-0.11	-0.45
CCSD[a]	-4113.12846	-4113.11194	-0.45	-4261.00069	-4260.98485	-4260.98153	-0.09	-0.43
CCSD(T)[a]	-4113.26790	-4113.25040	-0.48	-4261.15881	-4261.14240	-4261.13799	-0.12	-0.45
MR-CCSD[a]	-4113.12870	-4113.11291	-0.43	[b]	-4260.98536	-4260.98242	-0.08	-
Experiment			-0.40				0.70-0.76 (sum)	

[a] all CCSD calculations performed are DLPNO.
[b] calculation cannot be performed even providing 200+GB RAM per core.

Table S3. Total and relative energies of post-HF methods related to Table 1. (Dicopper tetraacetate).

Method	Tetraacetate+H_2O			Tetraacetate+$2H_2O$		
	Complex	Separate	E_b[eV]	Complex	Separate	E_b[eV]
CASSCF	-4263.469744	-4263.450155	-0.53	-4339.545731	-4339.507980	-1.03
DCD-CAS(2)	-4268.047299	-4268.023426	-0.65	-4344.382924	-4344.336338	-1.27
NEVPT2	-4268.047179	-4268.023288	-0.65	-4344.382820	-4344.336200	-1.27
SOS-MP2	-4267.754650	-4267.732483	-0.60	-4344.083875	-4344.040748	-1.17
DLPNO-CCSD	-4267.993660	-4267.971420	-0.61	-4344.334375	-4344.291062	-1.18
DLPNO-CCSD(T)	-4268.152883	-4268.130094	-0.62	-4344.501014	-4344.456556	-1.21
**Method**	**Tetraacetate+CO_2**			**Tetraacetate+$2CO_2$**		
	**Complex**	**Separate**	**E_b[eV]**	**Complex**	**Separate**	**E_b[eV]**
CASSCF	-4375.112075	-4375.105673	-0.17	-4562.831597	-4562.819013	-0.34
DCD-CAS(2)	-4380.036237	-4380.025242	-0.30	-4568.361862	-4568.340001	-0.59
NEVPT2	-4380.036148	-4380.025136	-0.30	-4568.361741	-4568.339892	-0.59
SOS-MP2	-4379.718768	-4379.709662	-0.25	-4568.013110	-4567.995103	-0.49
DLPNO-CCSD	-4379.967165	-4379.957247	-0.27	-4568.282872	-4568.262791	-0.55
DLPNO-CCSD(T)	-4380.145959	-4380.135625	-0.28	-4568.488612	-4568.467725	-0.57
**Method**	**Tetraacetate+CO**			**Tetraacetate+2CO**		
	**Complex**	**Separate**	**E_b[eV]**	**Complex**	**Separate**	**E_b[eV]**
CASSCF	-4300.173300	-4300.176689	0.09	-4412.962653	-4412.961000	-0.04
DCD-CAS(2)	-4304.858827	-4304.849309	-0.26	-4418.010815	-4417.988380	-0.61
NEVPT2	-4304.858512	-4304.849280	-0.25	-4418.010795	-4417.988070	-0.61

SOS-MP2	-4304.557649	-4304.552358	-0.14	-4417.696744	-4417.680495	-0.44
DLPNO-CCSD	-4304.798712	-4304.794095	-0.13	-4417.952026	-4417.936432	-0.42
DLPNO-CCSD(T)	-4304.967600	-4304.961861	-0.16	-4418.137790	-4418.120107	-0.48
**Method**	**Tetraacetate+O_2**			**Tetraacetate+2O_2**		
	**Complex**	**Separate**	**E_b[eV]**	**Complex**	**Separate**	**E_b[eV]**
CASSCF[a]	-4337.050277	-4337.050194	0.00	-4486.709042	-4486.708057	-0.03
DCD-CAS(2)[a]	-4341.846242	-4341.843374	-0.08	-4491.982227	-4491.976198	-0.16
NEVPT2[a]	-4341.846135	-4341.843232	-0.08	-4491.982127	-4491.976089	-0.16
SOS-MP2[b]	-4341.507810	-4341.505972	-0.05	-4491.588175	-4491.587724	-0.01
DLPNO-CCSD[c]	-4341.770493	-4341.767064	-0.09	-4491.888217	-4491.882388	-0.16
DLPNO-CCSD(T)	-4341.941498	-4341.937590	-0.11	-4492.078464	-4492.071581	-0.19
[a] Calculated at geometry "PBE+0.7" [b] Calculated at geometry "PBE+0.5" [c] Calculated at geometry "PBE+0.2"						
**Method**	**Tetraacetate+C_2H_4**			**Tetraacetate+2C_2H_4**		
	**Complex**	**Separate**	**E_b[eV]**	**Complex**	**Separate**	**E_b[eV]**
CASSCF	-4265.464444	-4265.456665	-0.21	-4343.536297	-4343.525615	-0.29
DCD-CAS(2)	-4270.119061	-4270.099069	-0.54	-4348.525597	-4348.483761	-1.14
NEVPT2	-4270.119033	-4270.09904	-0.54	-4348.52558	-4348.483742	-1.14
SOS-MP2	-4269.83499	-4269.819166	-0.43	-4348.24452	-4348.21285	-0.86
DLPNO-CCSD	-4270.082254	-4270.066846	-0.42	-4348.511648	-4348.479804	-0.87
DLPNO-CCSD(T)	-4270.248988	-4270.232512	-0.45	-4348.693215	-4348.658653	-0.94
**Method**	**Tetraacetate+CH_3OH**			**Tetraacetate+2CH_3OH**		
	**Complex**	**Separate**	**E_b[eV]**	**Complex**	**Separate**	**E_b[eV]**
CASSCF	-4302.503674	-4302.483168	-0.56	-4417.612612	-4417.574002	-1.05
DCD-CAS(2)	-4307.243538	-4307.217845	-0.70	-4422.775112	-4422.725088	-1.36
NEVPT2	-4307.243479	-4307.217705	-0.70	-4422.775079	-4422.725031	-1.36
SOS-MP2	-4306.954756	-4306.930588	-0.66	-4422.483329	-4422.436954	-1.26
DLPNO-CCSD	-4307.203317	-4307.179669	-0.64	-4422.753591	-4422.707589	-1.25
DLPNO-CCSD(T)	-4307.369305	-4307.345085	-0.66	-4422.933741	-4422.886570	-1.28
**Method**	**Tetraacetate+C_2H_5OH**			**Tetraacetate+2C_2H_5OH**		
	**Complex**	**Separate**	**E_b[eV]**	**Complex**	**Separate**	**E_b[eV]**
CASSCF	-4341.556581	-4341.541877	-0.40	-4495.718640	-4495.686050	-0.89
DCD-CAS(2)	-4346.467843	-4346.434953	-0.89	-4501.223175	-4501.163172	-1.63
NEVPT2	-4346.467724	-4346.434833	-0.90	-4501.223074	-4501.163051	-1.63
SOS-MP2	-4346.177774	-4346.150385	-0.75	-4500.929302	-4500.877782	-1.40
DLPNO-CCSD	-4346.438198	-4346.409848	-0.77	-4501.222875	-4501.169948	-1.44
DLPNO-CCSD(T)	-4346.611327	-4346.581340	-0.82	-4501.417312	-4501.361875	-1.51

Table S4. Total (upper part, Hartree) and relative (bottom part, eV) energies of O_2-PW-O_2 rigid scan shown on Figure 4. Addendum (in z-direction) is given in respect to PBE-geometry. Addendum of 20Åwas taken as full separation (zero interaction).

Add(Å)	CASSCF	NEVPT2	SOSMP2	MP2	DLPNO-CCSD	DLPNO-CCSD(T)
0.0	-4486.703460	-4491.965787	-4491.588175	-4491.945739	-4491.888217	-4492.078464
0.2	-4486.707387	-4491.978970	-4491.591299	-4491.948795	-4491.889818	-4492.079895
0.5	-4486.708930	-4491.982226	-4491.592264	-4491.949984	-4491.889093	-4492.078998
0.7	-4486.709042	-4491.982089	-4491.592067	-4491.949925	-4491.888361	-4492.078127
1.0	-4486.708847	-4491.981069	-4491.591349	-4491.949294	-4491.886971	-4492.076591
1.5	-4486.708351	-4491.979016	-4491.589871	-4491.947736	-4491.884636	-4492.074080
2.0	-4486.707988	-4491.977645	-4491.588732	-4491.946452	-4491.883255	-4492.072640
5.0	-4486.707610	-4491.975981	-4491.587461	-4491.944913	-4491.881776	-4492.071090
20.0	-4486.708057	-4491.976089	-4491.587724	[a]	-4491.882388	-4492.071581

[a] We found out that MP2 in ORCA4.2.1 has a bug, that produce unreasonable energies when parts of the molecule separated on high distance (difference get observable over 13Å). More details about issue can be found in https://orcaforum.kofo.mpg.de/viewtopic.php?f=8&t=6888&p=29634#p29639 For this reason Addendum of 5Å to PBE geometry (that corresponds to Cu-O distance of over 8Å) is taken as maximal separation.

Add(Å)	CASSCF	NEVPT2	SOSMP2	MP2	DLPNO-CCSD	DLPNO-CCSD(T)
0.0	0.13	0.28	-0.01	-0.02	-0.16	-0.19
0.2	0.02	-0.08	-0.10	-0.11	-0.20	-0.23
0.5	-0.02	-0.17	-0.12	-0.14	-0.18	-0.20
0.7	-0.03	-0.16	-0.12	-0.14	-0.16	-0.18
1.0	-0.02	-0.14	-0.10	-0.12	-0.14	-0.14
1.5	-0.01	-0.08	-0.06	-0.08	-0.08	-0.07
2.0	0.00	-0.04	-0.03	-0.04	-0.04	-0.03
5.0	0.01	0.00	0.01	0.00	0.02	0.01

Abbreviations

The following abbreviations are used in this manuscript:

B3LYP	Becke 3 parameter Lee-Yang-Parr functional
CCSD(T)	Coupled Cluster Singles, Doubles (and perturbational Triples)
DFT	Density Functional Theory
DLPNO	Domain-based Local Pair Natural Orbital
D3BJ	D3 dispersion correction with Becke-Johnson damping
E_b	Binding Energy
GB	GigaByte
GGA	Generalized Gradient Approximation
HOMO	Highest Occupied Molecular Orbital
I/O	Input/Output
LUMO	Lowest Unoccupied Molecular Orbital
MR	Multi-reference
M06L	Minnesota 2006 Local functional
PBE	Perdew-Burke-Ernzerhof functional
PW	Paddle-Wheel
RAM	Random-Access Memory
TB	TeraByte

References

1. Frisch, M.J.; Trucks, G.W.; Schlegel, H.B.; Scuseria, G.E.; Robb, M.A.; Cheeseman, J.R.; Scalmani, G.; Barone, V.; Petersson, G.A.; Nakatsuji, H.; Li, X.; Caricato, M.; Marenich, A.V.; Bloino, J.; Janesko, B.G.; Gomperts, R.; Mennucci, B.; Hratchian, H.P.; Ortiz, J.V.; Izmaylov, A.F.; Sonnenberg, J.L.; Williams-Young, D.; Ding, F.; Lipparini, F.; Egidi, F.; Goings, J.; Peng, B.; Petrone, A.; Henderson, T.; Ranasinghe, D.; Zakrzewski, V.G.; Gao, J.; Rega, N.; Zheng, G.; Liang, W.; Hada, M.; Ehara, M.; Toyota, K.; Fukuda, R.; Hasegawa, J.; Ishida, M.; Nakajima, T.; Honda, Y.; Kitao, O.; Nakai, H.; Vreven, T.; Throssell, K.; Montgomery, Jr., J.A.; Peralta, J.E.; Ogliaro, F.; Bearpark, M.J.; Heyd, J.J.; Brothers, E.N.; Kudin, K.N.; Staroverov, V.N.; Keith, T.A.; Kobayashi, R.; Normand, J.; Raghavachari, K.; Rendell, A.P.; Burant, J.C.; Iyengar, S.S.; Tomasi, J.; Cossi, M.; Millam, J.M.; Klene, M.; Adamo, C.; Cammi, R.; Ochterski, J.W.; Martin, R.L.; Morokuma, K.; Farkas, O.; Foresman, J.B.; Fox, D.J. Gaussian~16 Revision C.01, 2016. Gaussian Inc. Wallingford CT.
2. Perdew, J.P.; Burke, K.; Ernzerhof, M. Generalized Gradient Approximation Made Simple [Phys. Rev. Lett. 77, 3865 (1996)]. *Phys. Rev. Lett.* **1997**, *78*, 1396–1396. doi:10.1103/PhysRevLett.78.1396.
3. Becke, A.D. Density-functional thermochemistry. III. The role of exact exchange. *The Journal of Chemical Physics* **1993**, *98*, 5648–5652. doi:10.1063/1.464913.
4. Zhao, Y.; Truhlar, D.G. A new local density functional for main-group thermochemistry, transition metal bonding, thermochemical kinetics, and noncovalent interactions. *The Journal of Chemical Physics* **2006**, *125*, 194101. doi:10.1063/1.2370993.
5. Grimme, S.; Antony, J.; Ehrlich, S.; Krieg, H. A consistent and accurate ab initio parametrization of density functional dispersion correction (DFT-D) for the 94 elements H-Pu. *The Journal of Chemical Physics* **2010**, *132*, 154104. doi:10.1063/1.3382344.
6. Grimme, S.; Ehrlich, S.; Goerigk, L. Effect of the damping function in dispersion corrected density functional theory. *Journal of Computational Chemistry* **2011**, *32*, 1456–1465. doi:10.1002/jcc.21759.
7. Weigend, F.; Ahlrichs, R. Balanced basis sets of split valence, triple zeta valence and quadruple zeta valence quality for H to Rn: Design and assessment of accuracy. *Phys. Chem. Chem. Phys.* **2005**, *7*, 3297. doi:10.1039/b508541a.
